# A Novel Rolling Bearing Fault Diagnosis Method Based on the NEITD-ADTL-JS Algorithm

**DOI:** 10.3390/s25030873

**Published:** 2025-01-31

**Authors:** Shi Zhuo, Xiaofeng Bai, Junlong Han, Jianpeng Ma, Bojun Sun, Chengwei Li, Liwei Zhan

**Affiliations:** 1 Aero Engine Corporation of China Harbin Bearing Company, Ltd., Harbin 150500, China; zhuoshi8125@163.com (S.Z.); sunbjhit@163.com (B.S.); zhanliwei333@163.com (L.Z.); 2 School of Instrument Science and Engineering, Harbin Institute of Technology, Harbin 150001, China; 22b901042@stu.hit.edu.cn (X.B.); chengweili@hit.edu.cn (C.L.); 3 School of Automotive and Transportation Engineering, Shenzhen Polytechnic University, Shenzhen 518055, China

**Keywords:** rolling bearing, signal decomposition, NEITD-ADTL-JS, fault diagnosis

## Abstract

This paper proposes an innovative bearing fault diagnosis method aimed at enhancing the accuracy and effectiveness of transfer learning. The innovation lies in the signal preprocessing stage, where a Noise Eliminated Intrinsic Time-Scale Decomposition (NEITD) algorithm is introduced. This algorithm adaptively decomposes unified-phase sine wave signals to effectively extract the geometric mean of the intrinsic rotational component, and selects the optimal decomposition result based on the orthogonality index, significantly improving the quality and reliability of the signals. In addition, fault diagnosis parameters are adaptively optimized using an improved adaptive deep transfer learning (ADTL) network combined with the Jellyfish Search (JS) algorithm, further enhancing diagnostic performance. By innovatively combining signal noise reduction, feature extraction, and deep learning optimization techniques, this method significantly improves fault diagnosis accuracy and robustness. Comparative simulations and experimental analyses show that the NEITD algorithm outperforms traditional methods in both signal decomposition performance and diagnostic accuracy. Furthermore, the NEITD-ADTL-JS method demonstrates stronger sensitivity and recognition capabilities across various fault types, achieving a 5.29% improvement in accuracy.

## 1. Introduction

Rolling bearings are critical components in rotating machinery systems. Harsh operating environments greatly increase the risk of bearing failure [1,2], which can cause mechanical accidents and significant economic losses. Therefore, effective maintenance and health management require timely and accurate diagnosis of faults [3]. By monitoring and analyzing bearing vibration signals during operation, fault characteristics can be precisely extracted early on, enabling warning and prediction to effectively reduce potential hazards [4,5].

In traditional bearing vibration fault diagnosis techniques, fault features are typically extracted through time-frequency transformation [6]. However, actual vibration signals often contain significant noise components, making noise elimination during signal processing a major challenge. To address the issue of incomplete noise removal, Huang et al. [7] introduced a method called Empirical Mode Decomposition (EMD). This technique can automatically decompose the signal into Intrinsic Mode Functions (IMFs), offering the advantages of simplicity and speed. EMD has been widely applied in mechanical fault diagnosis [8,9], signal filtering [10], and other areas. Additionally, Smith proposed a method called Local Mean Decomposition (LMD), which defines the envelope based on the extrema and uses sliding mean interpolation instead of the cubic spline interpolation used in EMD [11]. However, this method exhibits endpoint effects similar to those of EMD. Dragomiretskii [12] developed a variant mode decomposition (VMD) model, which determines the bandwidth of each mode through an iterative search. Although this method can prevent mode mixing and improve decomposition performance, the selection of decomposition layers and associated penalty factors imposes certain limitations on performance. Feri et al. [13] proposed a method called Intrinsic Time Scale Decomposition (ITD), which, compared to EMD and its evolutionary algorithms LMD and VMD, offers significant advantages in terms of shorter processing time and more meaningful signal components.

Researchers have proposed various methods to further optimize the Intrinsic Time-Scale Decomposition (ITD) approach [14,15,16,17,18]. Yu et al. [19] utilized the ITD method to extract rotational components. Subsequently, these selected components were employed for signal reconstruction and fault diagnosis. Sheng et al. [20] introduced an improved support vector machine model, integrating ITD to process non-stationary features in bearing vibration signals. Duan et al. [21] developed a method for diagnosing gearbox faults under different operating conditions by combining hierarchical time memory with ITD. Feng et al. [22] proposed a denoising technique that combines improved singular value decomposition with ITD and integrates an enhanced deep residual network (ResNet) to achieve early detection of bearing faults. Guo et al. [23] introduced a method that fuses ITD, Hjorth parameters, and the Teager energy spectrum to effectively extract bearing fault features. Yu et al. [24] designed a fault feature enhancement method by combining exponentially weighted moving average with ITD. Additionally, Yu et al. [25] successfully extracted fault features of aviation bearings by integrating the autocorrelation function with ITD. Pan et al. [26], on the other hand, extracted bearing fault features by combining ITD with singular value decomposition (SVD).

To mitigate the impact of human factors, the application of deep learning methods for fault diagnosis has become increasingly prevalent [27,28,29,30]. Among these approaches, convolutional neural networks (CNNs) and their enhanced variants are widely employed [31,32,33,34,35]. However, to achieve greater practical applicability, it is essential to ensure that the training and test datasets share identical distributions. The advent of transfer learning methods has significantly enhanced performance when handling training and test data with differing probability distributions. Consequently, transfer learning and its optimization algorithms have been broadly adopted in bearing fault diagnosis.

Xu et al. [36] proposed a “Two-Stage Fault Diagnosis Assisted by Digital Twin” method, which utilizes deep transfer learning to transfer a pre-trained diagnostic algorithm from virtual to physical space for real-time monitoring and maintenance prediction. However, this approach may introduce discrepancies between the virtual and physical environments, limiting accuracy and reliability in practical production settings. Ma et al. [37] developed a transfer learning convolutional neural network based on AlexNet to diagnose bearing faults by extracting features from two-dimensional time-frequency images, achieving faster training speeds and higher accuracy. Nevertheless, this conversion process can lead to information loss and distortion, particularly with low spectral resolution. Although numerous studies [38,39,40,41,42] have explored similar approaches, the diversity of testing platforms and varying usage conditions often require transfer learning methods to assume that the target domain aligns with the source domain, which is not always applicable for bearing fault diagnosis. Consequently, many current studies exhibit limitations in their applicability.

To enhance the accuracy and effectiveness of transfer learning in bearing fault diagnosis, this paper proposes a novel fault diagnosis method called NEITD-ADTL-JS. This method not only facilitates the transfer of discriminative knowledge across different fault signals but also enables the effective detection of new fault categories. Compared to other fault diagnosis methods, this paper contributes to the area as follows:

(1) A new noise-assisted signal decomposition method, called the Noise Eliminated Intrinsic Time-Scale Decomposition (NEITD) algorithm, is proposed. The decomposed sine wave with a unified phase is supplemented to the original signal as the geometric mean of the signal, and then the mixed signal is filtered using the ITD method to improve the signal-to-noise ratio.

(2) The parameters of the transfer learning network are optimized using the Jellyfish Search algorithm, and a joint model consisting of a one-dimensional residual network and a deep adversarial transfer learning network is established to adaptively learn invariant features from both the source and target domains. Furthermore, a multi-label classifier optimized by joint maximum mean discrepancy is trained to achieve two-stage classification, identifying both known and unknown faults, ultimately enabling bearing fault diagnosis.

The article is structured as follows: Section 2 presents the theory of the conventional approach. Section 3 presents the theory and methodology proposed in this study. Section 4 compares and validates both analog and measured signals. Finally, Section 5 provides a comprehensive conclusion.

## 2. Related Theories

### Jellyfish Search Algorithm (JS)

Jellyfish Search is a new optimization method suggested by Chou et al. in 2021 [43]. This approach emulates the foraging behavior of jellyfish in the ocean and incorporates both exploration and development processes to establish an effective model for optimization algorithms. The specific methodology is outlined as follows:

Step 1: Initialization. It is assumed that the size of the jellyfish population is $n_{pp}$ and the highest number of iterations is $Max_{iter}$, and the upper and lower bounds of the search space are $U_{b}$ and $L_{b}$, respectively. A logistic chaotic map is employed to initialize the population of jellyfish, and the logistic chaotic map is presented in Equation (1).

(1) $$X_{i+1}=\eta X_{i}\left(1-X_{i}\right),0\leq X_{0}\leq 1$$

where $X_{i}$ is the logistic chaotic score of the *i*-th jellyfish position, *i* has a value range of $\left\{1,2,\cdots ,n_{pop}\right\}$, and $X_{0}$ represents the initial score of the logistic mapping, $X_{0}\notin \left\{0,0.25,0.5,0.75,1\right\},\eta =4$.

Step 2: A time control mechanism is presented to simulate the movement of jellyfish in different periods. The specific formula of the time control function is:

(2) $$C\left(t\right)=\left|\left(1-\frac{1}{{\mathrm{Max}}_{iter}}\right)\times \left(2\times \mathrm{rand}\left(0,1\right)-1\right)\right|.$$

Step 3: Define the movement mode of the jellyfish. In nature, the movement of jellyfish is driven by the nutrients contained in the ocean’s currents. Therefore, if C(t)≥0.5, the jellyfish will move with the ocean current. Equation (3) presents the position update.

(3) $$X_{i}\left(t+1\right)=X_{i}\left(t\right)+\mathrm{rand}\left(0,1\right)\times \left(X_{best}-\beta \times \mathrm{rand}\left(0,1\right)\times \mu \right)$$

$X_{i}\left(t\right)$ denotes the location of the *i*-th jellyfish of the *t*-th generation, $X_{best}$ is the best location in the current jellyfish population, β represents the coefficient of the distribution, which is generally taken as 3, and μ is the average location of all jellyfish in the population.

When C(t)<0.5 and rand(0,1)>(1−C(t)), the jellyfish moves passively, and the position update formula is

(4) $$X_{i}\left(t+1\right)=X_{i}\left(t\right)+\gamma \times \mathrm{rand}\left(0,1\right)\times \left(U_{b}-L_{b}\right)$$

γ represents the motion coefficient, generally taken at 0.1. When C(t)<0.5 and rand(0,1)≤(1−C(t)), the jellyfish makes active movement, and the position update formula is

(5) $$X_{i}\left(t+1\right)=X_{i}\left(t\right)+\mathrm{rand}\left(0,1\right)\times \bar{Direction}$$

$\vec{Direction}$ is intra-population movement mode, namely, $\vec{Direction}=\left\{\begin{array}{c} X_{j}\left(t\right)-X_{i}\left(t\right),\\ \;f\left(X_{i}\right)\geq f\left(X_{j}\right)\\ X_{i}\left(t\right)-X_{j}\left(t\right),\\ \;f\left(X_{i}\right)<f\left(X_{j}\right) \end{array}\;\right.$ and $X_{j}\left(t\right)$ are the positions of jellyfish randomly selected from the *t* generation population. $\left(j\neq i\right),f\left(X_{i}\right)$, and $f\left(X_{j}\right)$ represent the fitness values of the *i* and *j* jellyfish, respectively.

**Figure 1:**
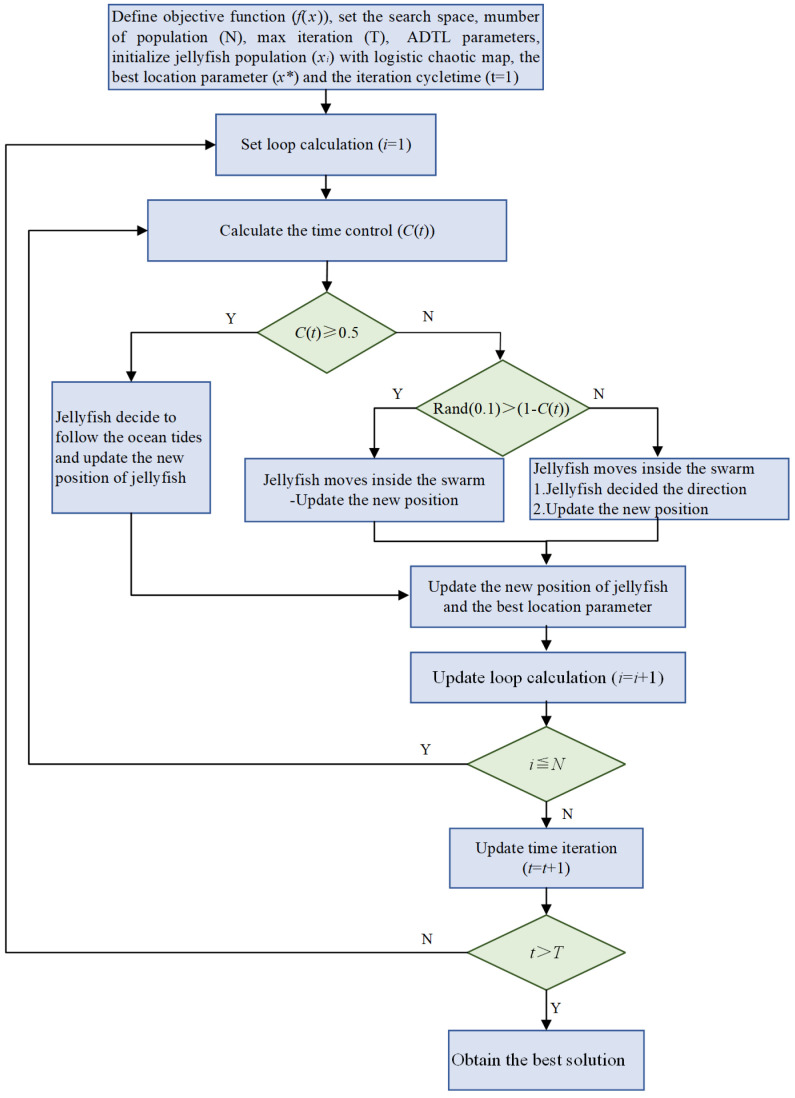
The Jellyfish Search algorithm flowchart.

The flowchart of the Jellyfish Search algorithm is shown in Figure 1.

## 3. Proposed Method

### 3.1. Noise Eliminated Intrinsic Time-Scale Decomposition Algorithm (NEITD)

Unlike traditional noise-assisted signal decomposition methods, the NEITD algorithm does not add white noise groups to the original signal before decomposition. Instead, it subtracts the geometric mean of the intrinsic rotation component (PRC) of the periodic ITD-decomposed adaptive-amplitude uniphase sine signal from the final intermediate frequency. To validate the effectiveness of the proposed approach, it is compared with ITD, EITD, and continuous wavelet transform. The results of analog signal decomposition show that the NEITD approach offers superior precision, sensitivity, and robustness.

The key steps of the NEITD algorithm are as follows: First, a set of adaptive-amplitude unified-phase sine wave signals is defined as the auxiliary decomposition signal. The frequency and phase of each signal are determined by the iteration period and signal length. Next, the generated auxiliary decomposition signal is added to the original signal to obtain a new signal. The upper and lower envelopes of the new signal are then extracted using the Hermite spline function. The average of these envelopes is calculated, and the signal is decomposed using the ITD method to obtain the PRC. The orthogonality index of each PRC is computed, and the component with the smallest orthogonality index is selected. Subsequently, ITD decomposition is applied to the auxiliary decomposition signal set, and the average PRC set is calculated. The average PRC obtained in this step is subtracted from the average PRC obtained from the auxiliary decomposition signal, resulting in a noise-free PRC. Finally, the first PRC is subtracted from the original signal, and the above steps are repeated for the remaining signal until the residual signal becomes monotonic or the number of extremum points drops below 3, thereby completing the final signal decomposition.

Compared to traditional decomposition methods, the NEITD algorithm demonstrates higher precision and robustness, particularly in noisy signal environments, significantly improving decomposition accuracy. Through adaptive amplitude adjustment and noise elimination, NEITD offers a more effective approach for signal decomposition, especially in scenarios with considerable noise interference. The pseudocode of Noise Eliminated Intrinsic Time-Scale Decomposition Algorithm are shown in Algorithm 1.
**Algorithm 1** Noise Eliminated Intrinsic Time-Scale Decomposition (NEITD)1:**Input:** Original signal x(t), amplitude ε, number of phase $n_{p}$, signal length *n*2:**Output:** Decomposed components $\left\{PRC_{j}\left(t\right)\right\}$ and residual r(t)3:Define $n_{prc}=\log_{2}\left(n\right)$4:**for** m=1 to $n_{prc}$ **do**5: $T_{w}=2^{m}$6: $f_{w}=1/T_{w}$7: **for** k=1 to $n_{p}$ **do**8:  $\theta_{k}=2\pi \left(k-1\right)/n_{p}$, $\varepsilon_{m}=\varepsilon \cdot r_{m-1}\left(t\right)$9:  Define auxiliary decomposition signal: $w\left(t;\varepsilon_{m};f_{w};\theta_{k}\right)=\varepsilon_{m}\cdot \cos\left(2\pi f_{w}t+\theta_{k}\right)$10:  Add to new signal: $x^{\prime }\left(t\right)=x\left(t\right)+w\left(t;\varepsilon_{m};f_{w};\theta_{k}\right)$11:  Perform ITD decomposition on $x^{\prime }\left(t\right)$ to obtain $PRC_{ij}\left(t\right)$12: **end for**13: Extract upper and lower envelopes of $x^{\prime }\left(t\right)$ using Hermite spline14: Calculate average envelope: $PRC_{j}\left(t\right)=\frac{1}{M}\sum_{i=1}^{M}PRC_{ij}\left(t\right)$15: Calculate orthogonality index for each $PRC_{j}\left(t\right)$16: Select component with smallest orthogonality index17: Perform ITD decomposition on auxiliary signals $w(t;\varepsilon_{m};f_{w};\theta_{k})$ to obtain $wc_{ij}\left(t\right)$ and residual $wr_{i}\left(t\right)$18: Compute average PRC for auxiliary signals: $wc_{j}\left(t\right)=\frac{1}{M}\sum_{i=1}^{M}wc_{ij}\left(t\right)$19: Calculate first PRC: $PRC_{j}=c_{j}\left(t\right)-wc_{j}\left(t\right)$20: Subtract first PRC from original signal: $x\left(t\right)=x\left(t\right)-PRC_{j}\left(t\right)$21:**end for**22:**While** residual signal is not monotonic and has more than 3 extrema points, **do** repeat steps 2–723:**Output**:$$x\left(t\right)=\sum_{i=1}^{M}PRC_{ij}\left(t\right)+rM_{j}\left(t\right)-wrM_{j}\left(t\right)$$

### 3.2. Adaptive Deep Transfer Learning Network-Jellyfish Search Algorithm (ADTL-JS)

**Figure 2:**
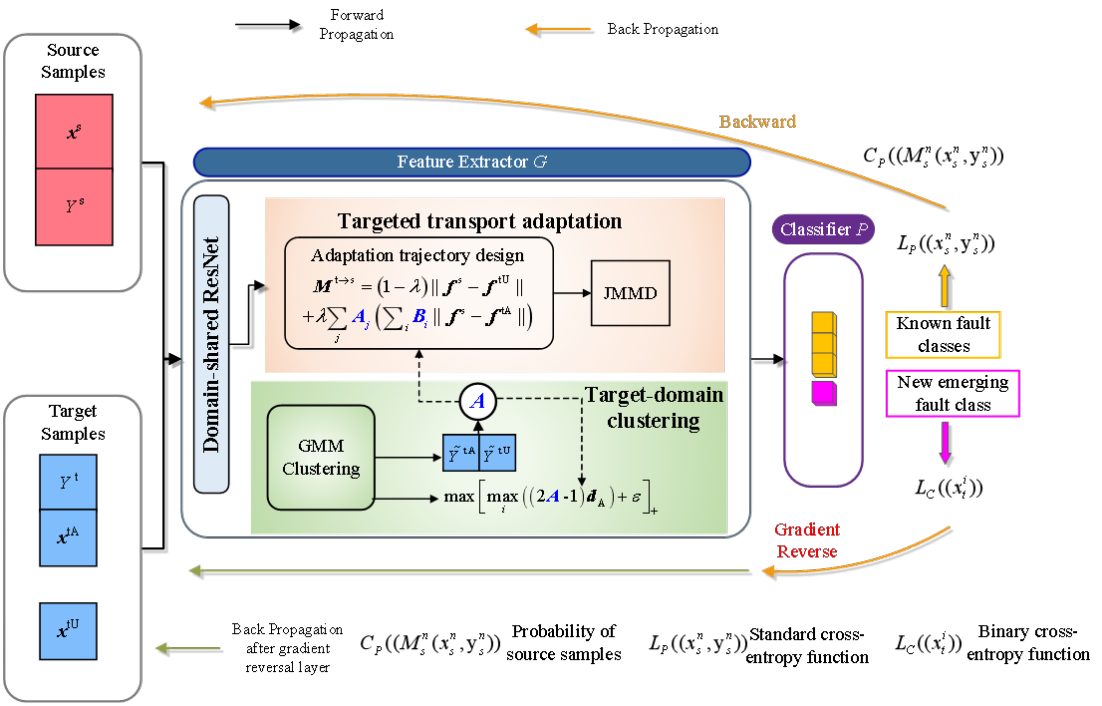
The suggested approach’s overall framework.

This article proposes a transfer learning method based on the ADTL-JS algorithm, which can not only complete the knowledge transfer of the mastered results in the source domain to the target domain but also judge the unknown faults in the target domain. To achieve the above functions, the transfer of known fault information is required, and the new fault types are carried out by utilizing the mastered results in the source domain. In the manuscript, the judgment of unknown faults is realized through a multi-layer classifier structure. Figure 2 depicts the suggested approach’s framework.

Step 1: Let the labeled source-domain samples, namely, the samples after noise reduction, be $X=\left\{\left(x_{s}^{N},y_{s}^{N}\right)\right\}$, where *N* denotes the source sample numbers, n∈{1,⋯,N} and $X_{Y}\in \left\{1,\cdots ,K\right\}$ are defined as the class labels of the source domain, and $X,T=\left\{\left(x_{t}^{I}\right)\right\}$ is the untagged samples in the target domain, where *I* represents the target sample numbers, i∈{1,⋯,I}.

Step 2: Build a domain-shared residual network to adaptively learn signal-invariant features. To extract features effectively, the algorithm proposed in the article uses a 1-dimensional residual network as a feature learner based on signal noise reduction and uses reconstructed signals for training. The standard convolutional layer consists of multiple convolutional kernels (filters), where the convolutional kernel’s size is usually smaller than the input graph, a setting that shapes a local receptive field. Furthermore, once each convolutional kernel slides over the input graph, the weights remain unchanged, which is called weight sharing. ReLU is selected as the activation function. The input of the BN layer is $H\in R^{m\times d}$, where *m* is the number of groups and *d* denotes the number of feature dimensions. The BN layer converts features i∈{1,⋯,d} into

(6) $$\begin{array}{c} {\hat{h}}_{i}=\frac{h_{i}-E\left[H_{.i}\right]}{\sqrt{\mathrm{Var}\left[H_{.i}\right]}}\\ O_{i}=\gamma_{i}{\hat{h}}_{i}+\beta_{i} \end{array}$$

where $h_{i}$ is the input scalar, $o_{i}$ is the output scalar, $H_{.i}$ is the *i*-th column of the input data, and $\gamma_{i}$ and $\beta_{i}$ are the values adapted by learning. At the end of the residual network, a fully connected softmax layer is usually employed to classify tasks. Assuming that the input data have *N* categories, the output of the softmax function is computed by Equation (7).

(7) $$\left[\begin{array}{c} p_{1}\left(x\right)\\ p_{2}\left(x\right)\\ \ldots \\ p_{N}\left(x\right) \end{array}\right]=\frac{1}{\sum_{N=1}^{N}e^{\theta^{\left(N\right)}x}}\left[\begin{array}{c} e^{\theta^{\left(1\right)}x}\\ e^{\theta^{\left(2\right)}x}\\ \cdots \\ e^{\theta^{\left(N\right)}x} \end{array}\right]$$

According to the softmax output, cross-entropy loss can be employed to gauge the error between the network output and the target label to optimize further.

**Figure 3:**
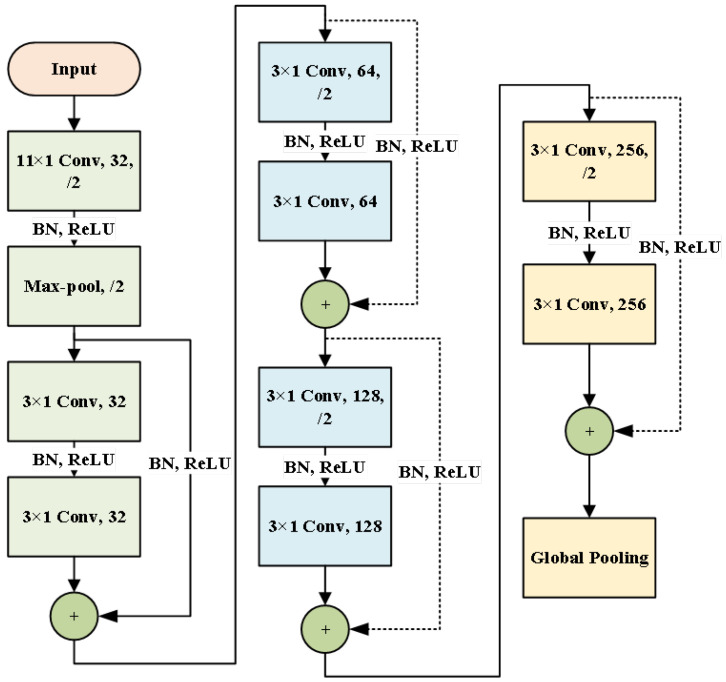
Structure diagram of residual network designed in this paper.

The network structure used is shown in Figure 3, including an initial convolution pooling layer and four residual learning layers, and enabled following the global pooling layer and classifier module for classification. Taking the initial convolution as an example, the parameters are described. The following Table 1 shows the network parameters. Moreover, a zero-filling scheme is run in all the convolution layers of the designed residual network. The filling size assigned to the initial convolution is 5, and the other filling sizes are 1. After multiple residual blocks, global pooling is designed to process the learned invariant attributes, which treats each feature graph as a region to perform pooling operations, and its output size is equal to the feature graph numbers. The neuron numbers in the classification layer are assigned to the identical number as the number of healthy classes.

Step 3: Train a multi-label classifier. Softmax regression is used to classify the mapping parameters of the invariant high-dimensional feature vector defined by the one-dimensional residual network. Considering the occurrence of unknown faults, the fault category numbers of the multi-label classifier trained in this study are set to K+1, so that the fault category numbers in its domain are the same as those in the target domain. For each target sample $\left(x_{t}^{i}\right)$, $p\left(y=K+1|x_{t}^{i}\right)$ is delineated as the new fault category’s probability. The probability of the source sample is defined as

(8) $$C_{P}=\left(M_{s}^{n}\left(x_{s}^{n},y_{s}^{n}\right)\right)=\left[\begin{array}{c} p\left(y=1|x_{s}^{n}\right)\\ p\left(y=2|x_{s}^{n}\right)\\ \vdots \\ p\left(y=K|x_{s}^{n}\right) \end{array}\right]=\frac{1}{\sum_{j=1}^{K}e^{zj}}\left[\begin{array}{c} e^{z1}\\ e^{z2}\\ e^{z3}\\ \vdots \\ e^{zK} \end{array}\right]$$

where $z_{j}=M_{s}\left(M_{s}^{n}\left(x_{s}^{n},y_{s}^{n}\right)\right)$ is the *j*-th value of $M_{s}^{n}\left(x_{s}^{n},y_{s}^{n}\right);e^{zj}$ denotes a normalization term; and $p\left(y=K|x_{s}^{n}\right)$ is the probability of a given fault class j∈{1,⋯,K}. The loss function is a standard cross-entropy function $L_{P}$.

(9) $$L_{P}\left(\left(x_{s}^{n},y_{s}^{n}\right)\right)=-\log\left[C_{P}\left(M_{s}^{n}\left(x_{s}^{n},y_{s}^{n}\right)\right)\right]$$

For unknown faults, a classifier is trained to distinguish known faults from unknown faults in the target samples, and the unknown fault samples are identified as new samples.

Step 4: Design the adaptive trajectory of the “unknown domain” and the source domain based on the adaptive definition of the joint maximum mean difference to realize the correction of the joint distribution between distinct domains and the minimization of the edge distribution offset. Initialize the relation matrix *A* between the unknown domain and the source domain. The binding labels of the samples in the unknown domain and the source domain determine the adaptive trajectory of the unknown domain. For the *j*-th sample, the L2 norm is used for calculation, as shown in Equation (10). The adaptive trajectory is shared with other target-domain samples with the same clustering label in the unknown domain, so that the elements in the relation matrix $M^{t\to s}\in R^{n_{s}\times n_{t}}$ are given in Equations (9) and (10).

(10) $$S_{j}=\frac{1}{\sum_{i}a_{ij}}\sum_{i=1}^{n_{s}^{\prime }}a_{ij}∥f_{i}^{s}-f_{k}^{tA}∥$$

The first term is the distance to correct the cross-domain sample features, the second term makes the unknown-domain samples move to the source-domain samples associated with the same label, and λ∈[0,1] denotes the trade-off parameter to tune the adaptive trajectory.

Calculate the joint maximum mean difference. To minimize the loss of the classification when the classification of the data in the source domain is processed, the optimization goal is to decrease the joint distribution distinction between the source and target domains. In this article, the Gaussian kernel function is picked to compute the ability of feature mapping to higher-dimensional space. The calculation method is as follows:

(11) $$L_{J}={\hat{J}}_{L}\left(P_{j},Q_{j}\right),L=\left\{f_{globalpool},f_{c}\right\}$$

(12) $$k\left(x,y\right)=\exp\left({∥x-y∥}^{2}/2\sigma^{2}\right)$$

(13) $$\begin{array}{cc} {\hat{J}}_{L}\left(P_{j},Q_{j}\right)= & \frac{1}{n_{s}^{2}}\sum_{i=1}^{n_{s}}\sum_{j=1}^{n_{s}}\prod_{l\in L}k^{l}\left(z_{i}^{sl},z_{j}^{sl}\right)\\ \; & +\frac{1}{n_{t}^{2}}\sum_{i=1}^{n_{t}}\sum_{j=1}^{n_{t}}\prod_{l\in L}k^{l}\left(z_{i}^{tl},z_{j}^{tl}\right)\\ \; & -\frac{1}{n_{s}n_{t}}\sum_{i=1}^{n_{s}}\sum_{j=1}^{n_{t}}\prod_{l\in L}k^{l}\left(z_{i}^{sl},z_{j}^{tl}\right) \end{array}$$

The suggested approach’s optimization objective is defined by

(14) $$\min_{\theta }J_{y}=\alpha w_{k}+\beta L_{J}$$

Step 5: Where θ is the training parameter of the one-dimensional residual network, α and β are the trade-off parameters, and the above parameters are adaptively optimized by using the Jellyfish Search algorithm.

The pseudocode of Noise Eliminated Intrinsic Time-Scale Decomposition Algorithm are shown in Algorithm 2.
**Algorithm 2** Adaptive Deep Transfer Learning Network-Jellyfish Search Algorithm (ADTL-JS)**Require:**1:Labeled source domain samples $X={\left\{\left(x_{s}^{n},y_{s}^{n}\right)\right\}}_{n=1}^{N}$2:Untagged target domain samples $T={\left\{x_{t}^{i}\right\}}_{i=1}^{I}$3:Number of classes *K*4:Trade-off parameters α, β5:Initialize jellyfish population $J=\{j_{1},j_{2},\cdots ,j_{P}\}$6:Initialize parameters θ, γ, β for the network**Ensure:**7:Trained ADTL-JS model8:**Build Domain-Shared Residual Network**9: Initialize residual network with convolution, BN, ReLU layers10: Define loss function $J_{y}=\alpha w_{k}+\beta L_{J}$11:**Train Multi-Label Classifier**12:**for** each epoch **do**13: **for** each batch $\left(x_{s}^{n},y_{s}^{n}\right)$ in *X* **do**14:  Forward pass through residual network to obtain $C_{P}$15:  Compute cross-entropy loss $L_{P}$16:  Backpropagate and update network parameters θ17: **end for**18:**end for**19:**Initialize Relation Matrix *A***20:Compute initial distances using L2 norm21:Assign adaptive trajectories based on clustering labels22:**Jellyfish Optimization Loop**23:**while** not converged **do**24: **for** each jellyfish $j_{p}$ in population *J* **do**25:  Update position and velocity based on jellyfish search behavior26:  Evaluate fitness using $J_{y}$27:   **end for**28: Select optimal jellyfish and update population *J*29: Update trade-off parameters α, β30:**end while**31:**Adaptive Trajectory and Distribution Alignment**32:**for** each target sample $x_{t}^{i}$ **do**33: Compute $p(y=K+1|x_{t}^{i})$ for unknown faults34: Assign to new fault category if probability exceeds threshold35:**end for**36:**Joint Maximum Mean Difference Calculation**37:Compute $L_{J}$ using Gaussian kernel for feature mapping38:Update network parameters to minimize $J_{y}$39:**Output Trained Model**40:**return** Trained ADTL-JS model

### 3.3. The Flow of the Proposed Algorithm

The fault diagnosis method proposed in this paper is as follows: First, original vibration signals from bearings with various fault types are collected using piezoelectric sensors. Then, the original data are labeled to define the source and target domains, where the source domain contains known fault categories, and the target domain includes both known and unknown fault categories. Next, the NEITD method is applied to the signals for noise reduction. NEITD excels in signal decomposition and noise suppression, effectively extracting valid components from complex signals, significantly improving signal quality. Furthermore, the combination with ADTL-JS enables the algorithm to address cross-domain migration issues, ensuring high diagnostic performance, especially when dealing with different operating conditions or unknown fault types.

Random samples are selected from both the source and target domains to construct a training dataset, with the remaining samples reserved for testing. Finally, the effectiveness of the ADTL-JS method is validated using all the test samples, and fault diagnosis results are automatically generated. By combining the NEITD, ADTL, and JS methods, we not only address noise interference and signal distortion in signal processing but also enhance the generalization ability of the algorithm, ensuring high diagnostic accuracy across various complex scenarios. The pseudocode of Noise Eliminated Intrinsic Time-Scale Decomposition Algorithm are shown in Algorithm 3.
**Algorithm 3** Fault Diagnosis Algorithm**Require:**$D_{\mathrm{original}}$: Original vibration signals collected from bearings$C_{\mathrm{known}}$: Set of known fault categories$C_{\mathrm{unknown}}$: Set of unknown fault categories**Ensure:**Fault diagnosis results1:**Step 1: Data Collection**2:Collect original vibration signals using piezoelectric sensors.3:$D_{\mathrm{original}}={\left\{x_{i}\right\}}_{i=1}^{N}$, where $x_{i}$ represents individual vibration signals.4:**Step 2: Data Labeling**5:Label the data to define source and target domains.6:$D_{\mathrm{source}}={\left\{x_{i},y_{i}\right\}}_{i=1}^{N_{s}}$ with $y_{i}\in C_{\mathrm{known}}$.7:$D_{\mathrm{target}}={\left\{x_{j},y_{j}\right\}}_{j=1}^{N_{t}}$ with $y_{j}\in C_{\mathrm{known}}\cup C_{\mathrm{unknown}}$.8:**Step 3: Noise Reduction and Dataset Construction**9:Apply Noise Reduction using NEITD method:10:${\tilde{x}}_{i}=\mathrm{NEITD}\left(x_{i}\right),\forall x_{i}\in D_{\mathrm{original}}$11:Randomly select samples for training:12:$D_{\mathrm{train}}=\mathrm{RandomSelect}\left({\tilde{D}}_{\mathrm{source}},{\tilde{D}}_{\mathrm{target}}\right)$13:$D_{\mathrm{test}}=D_{\mathrm{original}}\setminus D_{\mathrm{train}}$14:**Step 4: Fault Diagnosis Verification**15:Train ADTL model on $D_{\mathrm{train}}$:16:$\theta =\mathrm{ADTL\_Train}(D_{\mathrm{train}})$17:Predict fault types for $D_{\mathrm{test}}$:18:$\hat{Y}=\mathrm{ADTL\_Predict}\left(D_{\mathrm{test}},\theta \right)$19:Present fault diagnosis results automatically.20:**return** $\hat{Y}$

## 4. Experimental Results

### 4.1. The Analysis of Analog Signal

To assess the superiority of the proposed algorithm, various methods including NEITD, CEITDAN, and CEEMDAN decomposition are compared using numerical analog signals. The outcomes show that the suggested method presents leading performance regarding decomposition and noise reduction, thereby presenting significant potential for application in fault diagnosis. The analog signal employed in this study represents periodic impulse vibration signals generated when bearings experience localized damage due to multiple factors during prolonged operation. Signal analysis involves describing the signal as a convolution between an impulse signal and an impulse response signal. Let h(t) represent the impulse response signal and $A_{i}$ denote the circulating impulse signal. Considering the presence of noise interference in the actual project, where n(t) represents random noise, Equation (15) can be utilized to represent the analog signal.

(15) $$\begin{array}{c} x\left(t\right)=s\left(t\right)+n\left(t\right)=\sum_{i}A_{i}h\left(t-iT-\tau_{i}\right)+n\left(t\right)\\ A_{i}=A_{0}\cos\left(2\pi f_{r}t+\varphi_{A}\right)+C_{A}\\ h\left(t\right)=e^{-Ct}\sin\left(2\pi f_{n}t+\varphi_{m}\right) \end{array}$$

where s(t) represents the periodic fault shock; $f_{r}$ represents the rotation frequency $(f_{r}=$100Hz);C refers to the attenuation coefficient $\left(C=840\right);f_{n}$ denotes the resonance frequency $\left(f_{n}=3380\;\mathrm{Hz}\right)$; and $f_{i}$ represents the characteristic frequency of the inner-loop fault $\left(f_{i}=230\;\mathrm{Hz}\right).\tau_{i}$ denotes the slight sliding of the *i*-th shock in the period *T*, and after that, the sliding obeys the normal distribution with zero mean and 0.5% standard deviation of the frequency transition. n(t) denotes the random noise, whose SNR is −5 dB, while $f_{s}$ stands for the sampling frequency $\left(f_{s}=8192\;\mathrm{Hz}\right)$; 4096 data points are analyzed.

**Figure 4:**
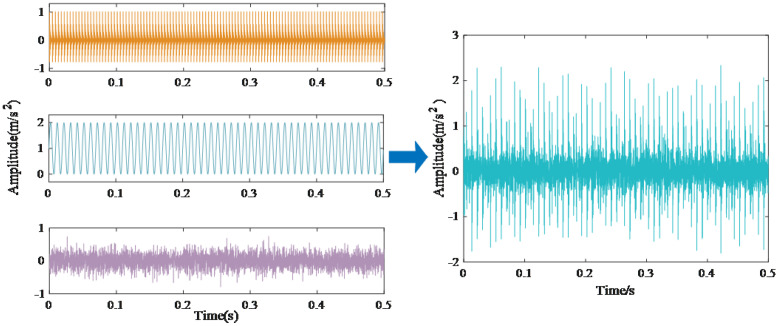
Time-domain diagram of analog signals.

The analog signals in Equation (15) effectively capture the intrinsic fault attributes of rolling bearings, thereby enabling a clear demonstration of the performance of the proposed algorithm. Figure 4 illustrates the time-domain representation of these analog signals utilized in this study.

**Figure 5:**
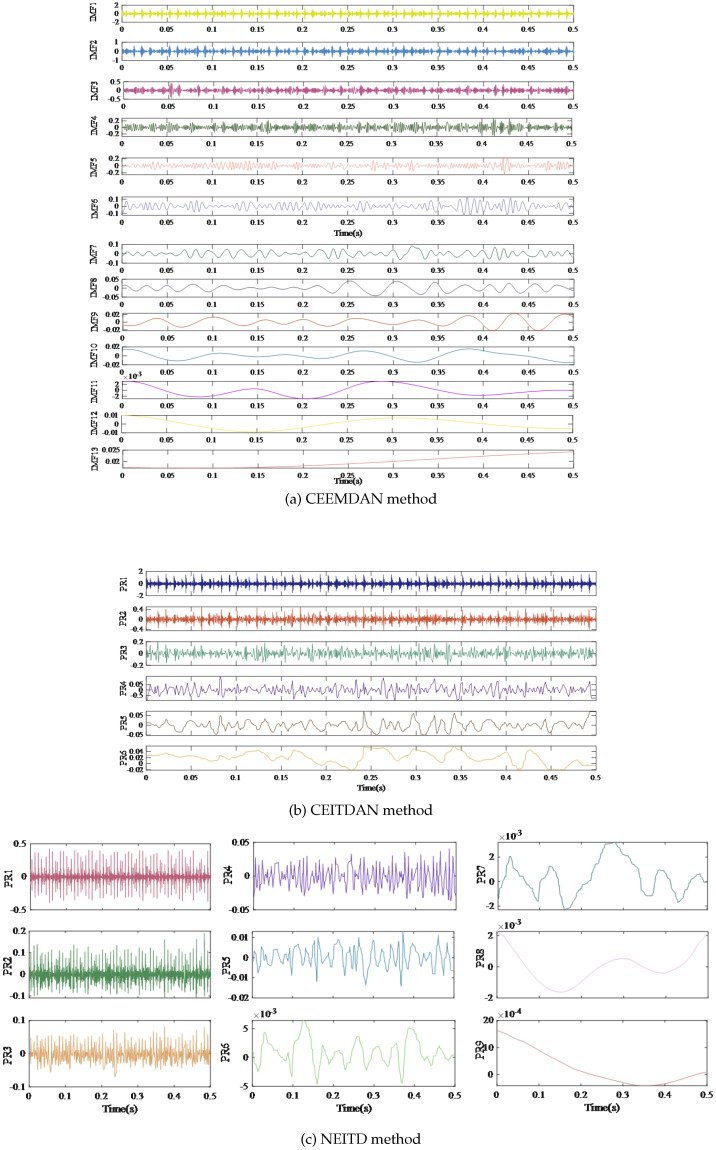
Decomposition results of different methods.

Figure 5 shows the decomposition results obtained by the three signal decomposition methods. The signal is disintegrated into 13 modal constituents by CEEMDAN, which is far more than the number of model components decomposed by NEITD and CEITDAN. Moreover, modal aliasing occurs in IMF 5 and IMF 6 when using CEEMDAN, while NEITD can efficiently resolve the issue.

To quantitatively assess the superiority of the proposed algorithm, we conducted a performance comparison between NEITD, CEEMDAN, and CEITDAN using several key metrics, including the orthogonality index [7]. The orthogonality index was chosen because it effectively measures the independence between components in the decomposition, thereby evaluating the accuracy with which low- and high-frequency signals are decomposed. In an ideal scenario, mode components derived from the NEITD, CEEMDAN, and CEITDAN methods should exhibit perfect orthogonality, resulting in an orthogonality index of 0. However, due to practical challenges such as environmental errors and signal interference, achieving a zero-orthogonality index is not feasible. Therefore, a lower orthogonality index reflects superior performance, indicating better decomposition accuracy and enhanced suppression of endpoint effects.

In addition to the orthogonality index, we also employed root mean square error (RMSE) and computational runtime as additional performance metrics. The RMSE was used to quantify the discrepancy between predicted and actual values, serving as an indicator of the decomposition’s accuracy and the signal’s standard error. The runtime metric evaluates the computational efficiency of the algorithms. A summary of these results is provided in Table 2.

**Figure 6:**
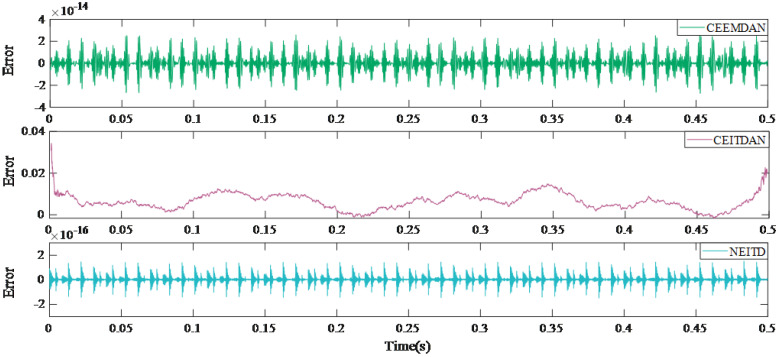
Reconstruction error of every method.

**Figure 7:**
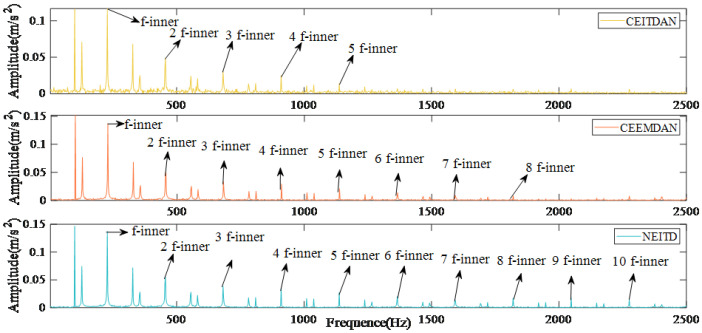
Results of envelope spectra of different methods.

The results of the reconstruction errors for different methods are presented in Figure 6. When compared to alternative algorithms, the proposed algorithm exhibits the lowest reconstruction error, measuring only $2\times 10^{-16}$. Figure 7 illustrates the envelope spectrum transformation results obtained using various methods. Although the CEITDAN approach is capable of deriving fault characteristic frequencies, there exists an issue with incomplete signal noise filtering. The CEEMDAN method reduces noise interference and improves the signal-to-noise ratio; however, beyond 1500 Hz, both fault information and noise are decomposed and filtered together due to weak fault signals being present. On the other hand, the proposed algorithm reduces noise interference while retaining fault characteristic information after 1500 Hz successfully. This paper verifies the proposed algorithm’s efficacy and superiority through objective quantitative indices as well as subjective characteristic frequency extraction.

**Figure 8:**
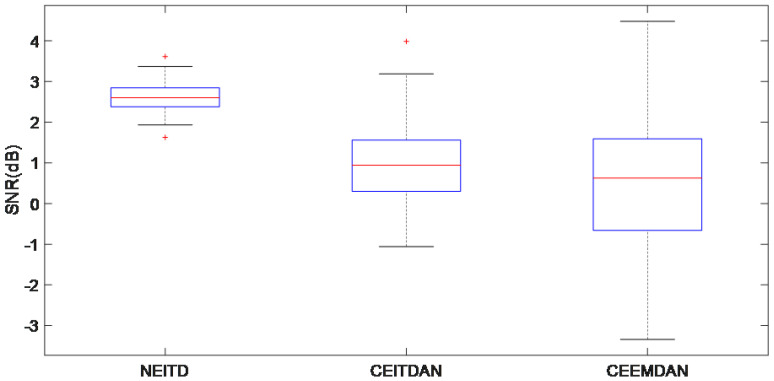
NEITD approach and comparison of various contrast methods in terms of their effectiveness in reducing noise.

The outcomes attained by employing the proposed algorithm and the approach at various SNR levels are summarized in Figure 8 using box plots. The suggested approach exhibits the biggest average SNR and minimal fluctuations. The box plots demonstrate the superior performance of the NEITD method compared to other approaches, thereby highlighting that utilizing estimated signal noise as auxiliary noise can significantly enhance the output signal’s SNR.

### 4.2. Test Signal Analysis

#### Public Dataset Validation

The training dataset utilized in the research was sourced from the open rolling bearing database of Case Western Reserve University. To simulate real-world scenarios with low signal-to-noise ratios and assess the algorithm’s robustness, random noise was introduced into the database. Experiments were conducted under four distinct conditions: 1797 rpm/0 hp (F0), 1772 rpm/1 hp (F1), 1750 rpm/2 hp (F2), and 1730 rpm/3 hp (F3). The accelerometer sampled fault data from the driving end bearing at a frequency of 12 kHz. The dataset encompasses four health phases, namely, normal condition (NC), inner-ring fault (IF), outer-ring fault (OF), and rolling element fault (RF). Each health phase exhibits varying degrees of severity. Overall, there are 10 health groups for each condition, comprising 1000 samples per category with a length of 1024, as illustrated in Table 3.

**Figure 9:**
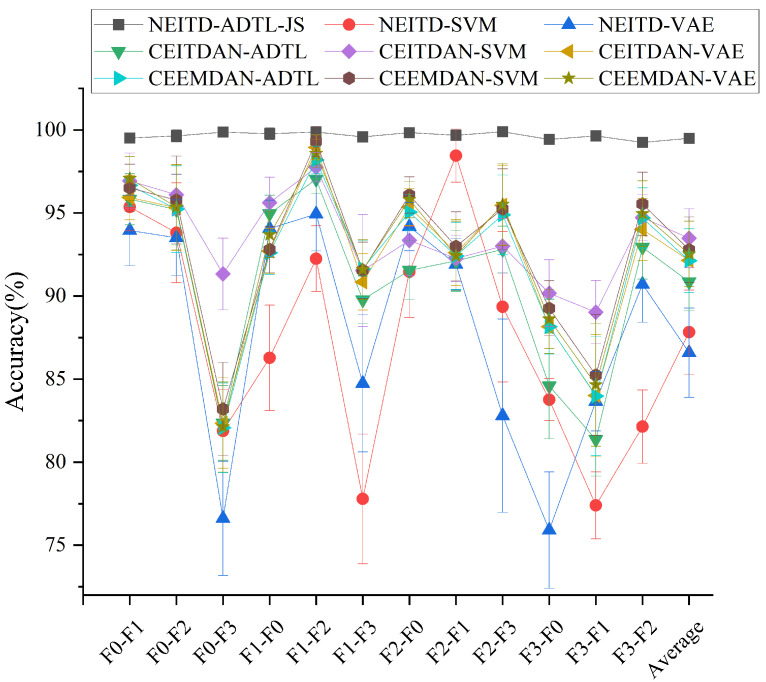
Classification accuracies of the different methods for bearing diagnosis tasks.

The presented results show the proposed algorithm’s leading performance in varied transfer tasks, thereby providing a highly effective classification capability. When compared to alternative techniques, the proposed algorithm consistently outperforms them and emphasizes the significance of domain adaptation for practical diagnostic applications. While certain methods like CEEMD-SVM achieve high average accuracy (e.g., 99.31% in tasks F1-F2), their performance exhibits significant variability across different tasks. Conversely, NEITD-ADTL-JS consistently delivers robust outcomes across diverse transfer tasks, as evidenced by an average accuracy of 99.50% shown in Figure 9, thus highlighting the suggested approach’s efficiency and superiority.

In addition to data analysis, the NEITD-ADTL-JS technology enhances performance evaluation through t-SNE visualization. The t-SNE visualization process first extracts features from the input data using different algorithms, which represent different categories or states. Then, the t-SNE algorithm reduces the high-dimensional features to two or three dimensions for visualization. By maintaining the relative distance between data points, t-SNE ensures that similar points are positioned close together in the low-dimensional space, thus revealing the distribution and relationships of categories. In the visualization results, different categories are typically represented by different colors or shapes, intuitively showing the clustering of data and the adaptability between the source and target domains. The t-SNE visualization effectively demonstrates the advantages of our algorithm in terms of class separation and domain adaptation, showing better clustering and adaptability compared to other methods.

**Figure 10:**
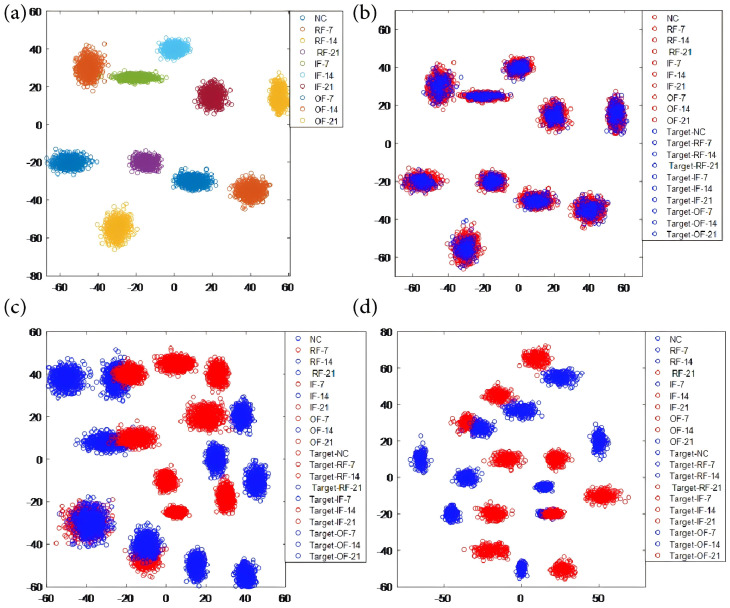
Visualization of t-SNE features. (**a**) NEITD-ADTL-JS; (**b**) CEEMDAN-SVM; (**c**) CEITDAN-VAE; (**d**) CEITDAN-SVM.

Given the low classification accuracy observed with methods such as NEITD-VAE, NEITD-SVM, and CEITDAN-ADTL, we provide visualization results for our algorithm and the CEEMDAN-SVM task (Figure 10a,b). These figures clearly illustrate how our method represents class information and domain information, respectively. Furthermore, Figure 10c,d showcase the domain adaptability of CEITDAN-VAE and CEITDAN-SVM. Specifically, in Figure 10a, we observe that our method excels in accurately classifying features and clustering those with the same health status; however, in Figure 10b,d, we notice that our method aligns the source- and target-domain features more closely, significantly outperforming the other two methods. This difference explains why the classification results of CEEMD-SVM and CEEMD-VAE are subpar. Therefore, these findings validate that NEITD-ADTL-JS not only excels at learning distinguishing features among classes for precise state recognition but also effectively bridges the domain gap.

### 4.3. Verification of Measured Data

**Figure 11:**
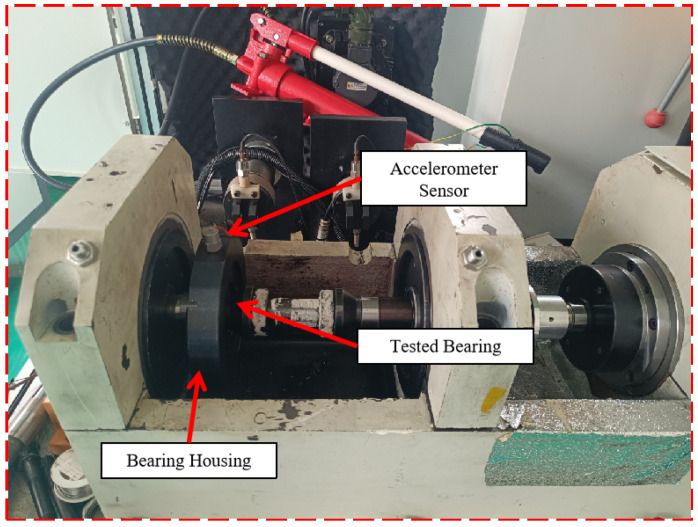
Test platform diagram.

To validate the proposed algorithm’s efficacy in practical engineering implementations, prefabricated fault data collected from an engineering simulation test bench for verification are employed, as depicted in Figure 11. The experimental design and comparative investigation included in this subsection are based on [44]. The test bench used in this study consists of a motor, shaft, coupling, tested bearing, bearing seat, and vibration sensor. It is primarily designed to simulate the bearing’s operational conditions, including varying speeds and loads. For this study, four distinct operating speeds were used: 3484 rpm, 4795 rpm, 6567 rpm, and 5784 rpm. The dataset used for analysis was generated from twelve different conversion scenarios, as presented in Table 4. Data were collected using a 50 kHz accelerometer, and the dataset encompasses four health phases: NC (normal condition), IF (inner fault), OF (outer fault), and RF (rolling fault), each representing varying degrees of fault severity.

The dataset includes 10 health categories, each consisting of 1000 samples with a length of 1024 for every operating condition, as presented in Table 4.

**Figure 12:**
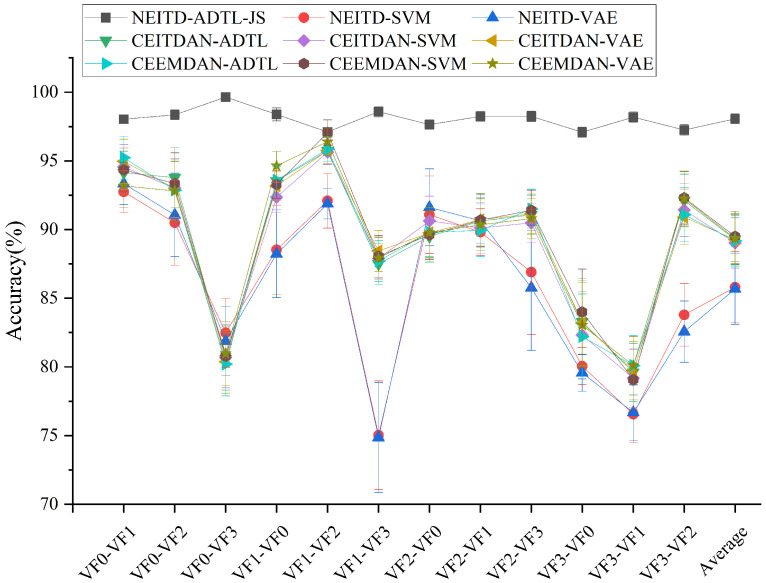
The algorithm suggested in this article is compared to the results of different approaches.

Figure 12 shows the leading classification outcomes of the proposed algorithm compared to other algorithms, particularly when dealing with a series of unknown faults and emerging faults. Among the five methods used for comparison, the proposed method consistently exhibits remarkable effectiveness. These findings underscore the crucial role of domain adaptation in meeting practical diagnostic application requirements. While some other comparative methods achieved average accuracies exceeding 95% in several tasks (e.g., CEITDAN-ADTL achieved an average accuracy of 95.73% in the VF1-VF2 tasks), their performances varied significantly across different tasks. Notably, accuracies in the VF0-VF3, VF3-VF0, and VF3-VF1 tasks were considerably lower than those observed in other tasks. Conversely, NEITD-ADTL consistently demonstrated robust results across various transmission tasks, thereby fully showcasing the reliability and adaptability of the proposed method. Consequently, these results not only validate the high efficiency of our approach, but also establish its dominant position within fault classification and domain adaptation research.

## 5. Conclusions

This paper proposes an innovative signal noise reduction method. The method combines the geometric mean of the rotating component, which is adaptively decomposed from the unified-phase sine wave signal obtained through the ITD method, with the original signal. The optimal rotating component is selected using the orthogonality index, effectively addressing the mode-aliasing problem present in ITD. Through this innovative signal processing technique, the signal decomposition capability and noise filtering effectiveness are significantly improved. Furthermore, to improve the accuracy of bearing fault diagnosis, this paper introduces an adaptive deep transfer learning network combined with the Jellyfish Search (JS) algorithm. This network adaptively learns shared features from both the source and target domains, effectively achieving cross-domain transfer and demonstrating excellent performance in various complex fault scenarios. Compared to traditional methods, the NEITD-ADTL-JS method can more accurately identify both known and unknown fault types, achieving a 5.29% improvement in accuracy.

## Figures and Tables

**Table 1:** Initial convolution parameters.

Size of the Convolution Kernel	Convolution Kernel Numbers	Step Size	Activation Function
11 × 1	32	2	ReLU

**Table 2:** Outcomes of using different algorithms to quantify indicators.

Method	IO	RMSE	Time (s)
NEITD	0.06	3.4197 × $10^{-17}$	0.5
CEEMDAN	0.23	1.5441 ×$10^{-14}$	15.3
CEITDAN	0.11	0.0001	2.1

**Table 3:** This article uses sample situations to illustrate.

Transfer Tasks	Source Domain	Target Domain	Health Conditions
F0-F1	1797 rpm/0 hp	1772 rpm/1 hp	NC, RF-7,
F0-F2	1797 rpm/0 hp	1750 rpm/2 hp	RF-14, RF-21,
F0-F3	1797 rpm/0 hp	1730 rpm/3 hp	IF-7, IF-14,
F1-F0	1772 rpm/1 hp	1797 rpm/0 hp	IF-21, OF-7,
F1-F2	1772 rpm/1 hp	1750 rpm/2 hp	OF-14, OF-21
F1-F3	1772 rpm/1 hp	1730 rpm/3 hp	
F2-F0	1750 rpm/2 hp	1797 rpm/0 hp	
F2-F1	1750 rpm/2 hp	1772 rpm/1 hp	
F2-F3	1750 rpm/2 hp	1730 rpm/3 hp	
F3-F0	1730 rpm/3 hp	1797 rpm/0 hp	
F3-F1	1730 rpm/3 hp	1772 rpm/1 hp	
F3-F2	1730 rpm/3 hp	1750 rpm/2 hp	

**Table 4:** Description of the rolling bearing diagnosis.

Transfer Tasks	Source Domain	Target Domain
VF0-VF1	3484 rpm	4795 rpm
VF0-VF2	3484 rpm	6567 rpm
VF0-VF3	3484 rpm	5784 rpm
VF1-VF0	4795 rpm	3484 rpm
VF1-VF2	4795 rpm	6567 rrpm
VF1-VF3	4795 rpm	5784 rpm
VF2-VF0	6567 rpm	3484 rpm
VF2-VF1	6567 rpm	4795 rpm
VF2-VF3	6567 rpm	5784 rpm
VF3-VF0	5784 rpm	3484 rpm
VF3-VF1	5784 rpm	4795 rpm
VF3-VF2	5784 rpm	6567 rpm

## Data Availability

Data are contained within the article.
